# Identification and validation of monocyte to macrophage differentiation-associated as a prognostic biomarker in gastric cancer

**DOI:** 10.3389/fonc.2025.1508355

**Published:** 2025-04-16

**Authors:** Suyang Bai, Zhaofeng Chen, Rui Ji, Yuping Wang, Yongning Zhou, Qinghong Guo, Liang Qiao

**Affiliations:** ^1^ The First Clinical Medical College, Lanzhou University, Lanzhou, China; ^2^ Department of Gastroenterology, The First Hospital of Lanzhou University, Lanzhou, China; ^3^ Gansu Province Clinical Research Center for Digestive Diseases, The First Hospital of Lanzhou University, Lanzhou, China; ^4^ Storr Liver Centre, The Westmead Institute for Medical Research (WIMR), The University of Sydney, Westmead, NSW, Australia

**Keywords:** MMD, stomach neoplasms, prognosis, biomarker, miR-200b-3p

## Abstract

**Background:**

Gastric cancer (GC) has a very poor prognosis as most cases are diagnosed at a late stage, which can be partially attributed to a lack of reliable diagnostic biomarkers. Our study reveals a close correlation between monocyte to macrophage differentiation-associated (MMD) and GC.

**Methods:**

We analyzed data from The Cancer Genome Atlas (TCGA). A close association between MMD levels and the clinicopathological features of gastric cancer patients was identified using Cox regression analysis and KM plot database analysis. Bioinformatics data were validated by real-time polymerase chain reaction and western blot analysis in GC cells. The impact of MMD on GC was examined using multiple complementary assays, including colony formation assay, CCK-8 assay, cell cycle analysis, apoptosis assessment, wound healing assay, transwell assay, and subcutaneous xenograft tumor formation assay in mice.

**Results:**

High levels of MMD were observed in GC tissues. MMD accelerated cell growth and metastasis, and suppressed apoptosis in GC cells. MMD inhibition significantly suppressed the growth of xenograft tumors in mice. Further studies had revealed that MMD expression was suppressed by miR-200b-3p in GC. Dual luciferase experiment indicated that MMD is a direct target gene of miR-200b-3p. MMD might play an oncogenic role in GC by acting as a direct target of miR-200b-3p.

**Conclusion:**

MMD plays an oncogenic role in gastric cancer. It may serve as a potential biomarker for GC diagnosis and a therapeutic target.

## 1 Introduction

Gastric cancer (GC), a prevalent malignancy, poses a significant global health risk. The incidence and mortality rates are consistently high. The latest global cancer epidemiology data (1) show that the global incidence of gastric cancer is 5.6%, placing it fifth in cancer occurrence, and it has a 7.7% mortality rate, ranking fourth for cancer deaths. In males, gastric cancer ranks fourth in incidence (7.1%) and third in mortality (9.1%), whereas in females, it ranks seventh in incidence (4.0%) and fifth in mortality (6.0%). The incidence of gastric cancer varies significantly across regions. Three critical areas—East Asia, Eastern Europe, and South America–exhibit notably higher incidences than others. Notably, Japan has the highest incidence of gastric cancer in men and Mongolia has the highest incidence of gastric cancer among women globally. Men generally have higher gastric cancer rates than women. Therefore, the search for new and more reliable markers could aid in the diagnosis and prognostic assessment of gastric cancer.

Monocyte to macrophage differentiation-associated (MMD), also known as PAQR11, is one of the progesterone and lipocalin molecule receptor family members. MMD is a novel rat sarcoma protein (Ras) modulator that activates Ras signaling in the Golgi complex (2). MMD is associated with macrophage activation, which may involve extracellular regulated protein kinases (ERK) and protein kinase B (AKT) phosphorylation (3). Literature has reported that MMD is essential for lung cancer cell migration in an epithelial-mesenchymal transition (EMT)-induced lung adenocarcinoma model (4). MMD modulates the ERK pathway, thereby influencing lung cancer cell growth (5). MMD also modulates the disease progression of rheumatoid arthritis in mice (6). MMD also modulates disease progression of rheumatoid arthritis in mice (6), and it regulates lipolysis and affects obesity (7). MMD suppresses rat microglial activation and inflammatory reaction post subarachnoid hemorrhage (8). A recent study showed that collaboration between MMD, acyl-CoA synthetase long chain family member 4 (ACSL4), and membrane bound O-acyltransferase domain containing 7 (MBOAT7) enhances the process of polyunsaturated phosphatidylinositol remodeling and increases vulnerability to iron-induced cell death in cancer cells (9). However, there have been no reports on the relationship between MMD and GC.

This study used The Cancer Genome Atlas (TCGA) and other databases to investigate MMD levels and their prognostic significance in gastric cancer. These enrichment and immune infiltration analyses provide a preliminary introduction to the possible biological role of MMD and its influence on the stomach cancer immune microenvironment. We confirmed MMD expression in GC and its impact on gastric cancer development. Therefore, we explored the possibility of using MMD as a GC biomarker.

## 2 Materials and methods

### 2.1 Data collection and collation

The TCGA_GTEx-STAD dataset and related clinical data were obtained from XENA (10). MMD levels in the GC tissues were assessed using the Wilcoxon rank-sum test. An analysis of MMD level and its clinical significance in GC was performed using the Kruskal–Wallis test, and the results were graphically represented using the “gglot2” package. RNA sequencing and microRNA (miRNA) sequencing data for gastric adenocarcinoma (TCGA-STAD) and clinical information were obtained and organized from the TCGA repository. The results were visualized by “gglot2” after analyzing the correlation between MMD and 18 miRNAs. The proportional risk hypothesis testing and Cox regression analysis based on the MMD expression data in TCGA-STAD RNAseq and the clinical data of patients were performed using the R package “survival”.

### 2.2 Database analysis

GEPIA2 (11) studied the impact of MMD expression on the survival of individuals with GC and its association with six markers of cancer-related fibroblasts. The association of MMD expression levels with prognostic survival time and time to first disease progression in GC patients was analyzed using the KM plot database (12), and the effects of high and low MMD expression in clinicopathological characterization subgroups of gastric cancer were further analyzed. TIMER 2.0 (13) was used to examine the association between MMD levels and immune cells in GC. Four databases, ENCORI, MicroT-CDS, TarBase, and TargetScanHuman8.0, were utilized to predict the upstream miRNAs of MMD, and TargetScan predicted the binding site of MMD to miR-200b-3p.

### 2.3 Functional enrichment analysis

A series of differentially expressed genes (DEGs) were identified between cohorts with low and high MMD expression using the TCGA dataset. The DEGs were chosen based on criteria, including more than one absolute value of log2 Fold Change and p-adj less than 0.05 (14). This study aimed to explore the biological significance of these genes and the pathways involved. First, the Gene Ontology/Kyoto Encyclopedia of Genes and Genomes (GO/KEGG) studies were performed to better understand the biological mechanisms associated with the DEGs. DEGs were analyzed for significant enrichment using gene set enrichment analysis (GSEA) (15). These analyses used the R packages “DESeq2” and “clusterProfiler” (14, 16).

### 2.4 Cell culture

The normal gastric epithelial cell line, GES-1, was acquired from the Gansu Key Laboratory of Gastroenterology at the First Hospital of Lanzhou University. Five cell lines derived from GC (HGC-27, AGS, MKN-45, SNU-216, and SNU-668) were acquired from the Guangzhou Saiku Biological Company. Five cell lines (GES-1, MKN-45, SNU-216, SNU-668, and HGC-27) were grown in RPMI 1640 (Viva Cell, China). AGS cells were grown in the F12K medium (BOSTER, China). Fetal bovine serum (FBS) (ABW, China) was added to the medium (medium: FBS=9:1). Cells were passaged 2 times after resuscitation for subsequent experiments. All cells were passaged no more than 15 times and passaged 2–3 times a week. All samples were placed in an incubator (37°C, 5% CO2).

### 2.5 Real-time quantitative PCR

Total RNA was isolated from cells using RNAiso Plus (TaKaRa). After adding 1/5 volume of chloroform to the lysate, the mixture was incubated at room temperature for 10 minutes. Subsequent centrifugation was performed at 12,000 × g for 15 minutes at 4°C. The aqueous phase containing RNA was carefully transferred, and an equal volume of isopropanol was added to precipitate nucleic acids. Following thorough mixing and 10-minute incubation at room temperature, samples were centrifuged at 12,000 × g for 10 minutes at 4°C. The resultant RNA was washed twice with 75% ethanol and air-dried briefly. Finally, RNA was resuspended in an appropriate volume of RNase-free water and quantified using ultraviolet spectrophotometry. Followed by reverse transcription to produce cDNA using the cDNA Synthesis Kit (AT311, TransGen, China) and miRNA Synthesis Kit (638313, TaKaRa), respectively. MMD mRNA expression was confirmed using TransStart Top Green qPCR SuperMix (AQ131, TransGen, China). MiRNA expression was validated using TB Green Premix Ex Taq II (RR820; TaKaRa, China). All qPCR experiments were replicated at least three independent times. These qPCR data were analyzed using the ΔΔCt method. **Supplementary Table 1** lists the qPCR primer sequences.

### 2.6 Western blotting

The total proteins of GC cells were isolated using RIPA buffer (Solarbio, China). The target proteins were isolated using 12% SDS-PAGE. Following transfer to the polyvinylidene fluoride (PVDF) membranes (0.45 µm), these membranes were blocked with skim milk (5%) (Beyotime, China) and subsequently exposed to the primary antibodies beta actin monoclonal antibody (66009-1-Ig, Proteintech, China) and MMD (E4U4G) Rabbit mAb (20226S, Cell Signaling Technology, USA), and the secondary antibodies, including anti-mouse antibody (7076, Cell Signaling Technology, USA) and anti-rabbit antibody (7074, Cell Signaling Technology, USA). Finally, chemiluminescence (SQ210L, Epizyme Biotech, China) was applied to the PVDF membrane and visualized using a gel phosphorimager (Monad, China).

### 2.7 Cell transfection

GenePharma (China) designed and synthesized the negative control (NC), MMD-siRNA-1, MMD-siRNA-2, inhibitor control, miR-200b-3p inhibitor, mimic control, and miR-200b-3p mimic. GeneChem (China) designed and synthesized MMD overexpression plasmids and control plasmids. The EndoFectin-Max Manual transfection reagent (EF013) was purchased from GeneCopoeia. Transfection experiments were conducted in six-well cell culture plates.

### 2.8 Cell proliferation

Following the alteration of MMD expression through cell transfection in a six-well plate, cells from the control and experimental groups were transferred to 96-well cell culture plates according to 3000 cells (100 µl) per well, with three wells per group for replication. At the correct moment, 10 µl Cell Counting Kit-8 reagent (Biosharp, China) was introduced into each well. The cells were plated in an incubator for a sufficient duration. Absorbance (A450) was assessed at 0, 24, 48, 72, and 96 h using an enzyme standard (Thermo Scientific, USA).

### 2.9 Clone formation assay

After cell transfection, single-cell suspensions were prepared from the control and experimental groups. Following cell enumeration, the correct cell quantity was transferred to fresh six-well dishes and incubated in a cell culture chamber for a week. The cells at the bottom of the wells were treated with paraformaldehyde (4%), stained with crystal violet (0.1%), and subsequently photographed.

### 2.10 Flow cytometry

Twenty-four hours after transfection, cells from the control and experimental groups were collected using the Cell Cycle Staining Kit (Multi Sciences, China) and Annexin V-APC/PI Apoptosis Detection Kit (KeyGen BioTech, China), respectively. Flow cytometry was used to analyze the cell cycle and apoptosis.

### 2.11 Subcutaneous xenograft experiment

MKN-45 cells were employed for lentiviral vector and MMD-shRNA (Hanbio, China) stable transplants, with puromycin (2 µg/ml) screening for MMD knockdown. Sixteen nude mice aged 4-6 weeks were purchased from GemPharmatech and divided into control and experimental groups (eight mice per group). Each nude mouse was injected subcutaneously with MKN-45 cells (5×10^6^ cells in 100 μl), and tumor size (long diameter and short diameter) was measured every 2 days with a caliper after tumor formation.

### 2.12 Wound healing assay

After cell transfection, when the cells were spread over the whole bottom of the wells, a vertical line was drawn in the middle of the bottom surface inside each well with a 200 μl pipette tip. Five lines perpendicular to the scratch were marked on the bottom of the plate, and the five points were photographed under a microscope at 0 and 24 h after scratching (objective lens, 10×).

### 2.13 Transwell assay

An 8 μm pore size chamber (#3422, Corning, USA) was utilized for cell migration and invasion assays. During migration, 600 μl of complete medium (AGS: 90% F12K and 10% FBS; SNU-216: 90% RPMI 1640 and 10% FBS) was introduced into the lower section of the chambers, and the upper chamber contained 200 μl serum-free single-cell suspensions with 5×10^4^ cells. Invasion experiments required pre-preparation of the matrix gel (#354234, BD Biosciences, USA) in the upper chamber at a specific dilution ratio (1:8 for AGS; 1:15 for SNU-216). The experimental protocol required an incubation period of 24-30 hours. Subsequently, the cells were treated with paraformaldehyde and stained with crystal violet. Finally, cells on the exterior of the chamber were observed under a microscope (objective lens 20×).

### 2.14 miRNA prediction

Four databases were used to predict the upstream regulatory miRNAs of MMD. The MicroT-CDS database predicted 203 miRNAs, the TargetScan database predicted 457 miRNAs, the ENCORI database predicted 160 miRNAs, and the TarBase database predicted 89 miRNAs. Subsequently, the prediction results of the four databases were intersected to obtain 18 miRNAs. Further analysis of the correlation between these 18 miRNAs and MMD expression in gastric cancer tissues revealed that miR-200b-3p had the highest correlation with MMD expression. Subsequently, the effect of miR-200b-3p on the prognosis of gastric cancer was analyzed, and the expression of miR-200b-3p was verified in gastric cancer cells.

### 2.15 Dual luciferase assay

The GP-CHECK2 (GenePharma, China) dual-luciferase reporter vector was used to integrate the MMD-miR-200b-3p-WT and MMD-miR-200b-3p-MUT sequences. These tools were co-transfected into 293T cells along with a control mimic (NC) and miR-200b-3p mimics, followed by 48 h of incubation under standard conditions. Cell lysates were collected using a dual-luciferase system (GenePharma, China) and then examined using a multimode reader (BioTek, USA).

### 2.16 Statistical analysis

The gray values of the bands in western blotting were calculated using ImageJ software (National Institutes of Health, USA). Finally, GraphPad Prism 9.0 (GraphPad Software, USA) was used to statistically analyze and plot the experimental data. A t-test was used to analyze the statistical significances of differences between two groups, and p<0.05 indicated statistical significance.

## 3 Results

### 3.1 MMD is highly expressed in GC

**Figure 1:**
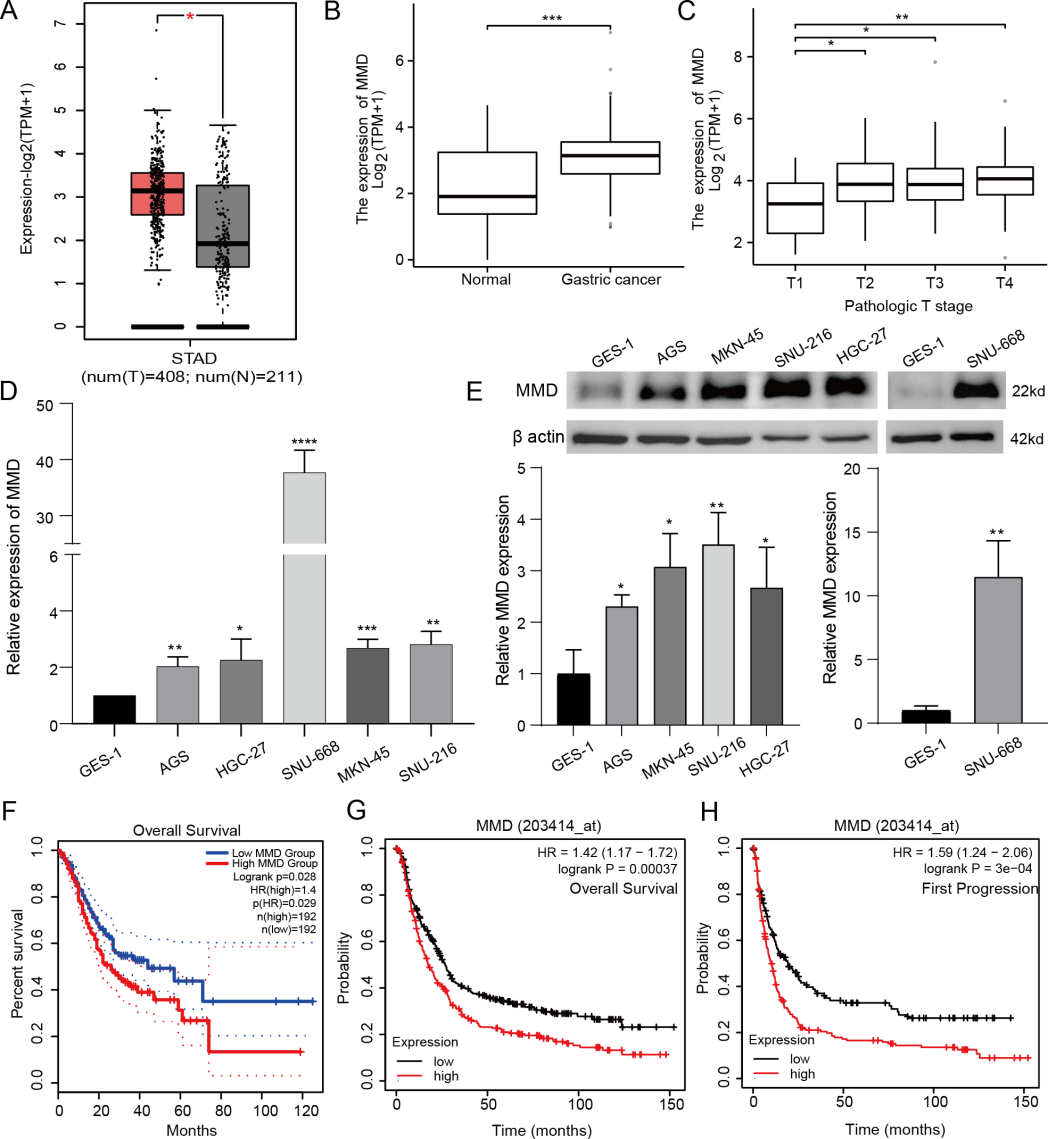
MMD expression and prognostic survival analysis. **(A, B)** MMD expression in GC tissues; **(C)** MMD and T-stage in GC; **(D, E)** MMD expression in GC cells (qPCR,western blotting); **(F–H)** Comparison of prognostic situations between groups with high and low MMD in GC patients. (*p<0.05, **p<0.01, ***p<0.001, ****p<0.0001).

We initially observed MMD expression in GC tissues in GEPIA2 (**Figure 1A**). Furthermore, examination of the TCGA_GTEx-STAD dataset from the XENA database revealed that MMD expression was notably increased in GC tissues compared with that in non-GC tissues (**Figure 1B**), including 174 Genotype-Tissue Expression database (GTEx) normal tissues, 36 TCGA-STAD paracancerous tissues, and 414 TCGA-STAD gastric cancer tissues.

MMD expression levels were measured in GES-1 and GC cell lines by qPCR and western blot analyses (**Figures 1D, E**). These findings indicated that MMD expression levels were increased in multiple gastric cancer cell lines.

### 3.2 MMD is associated with T-stage of GC

Using R software, analysis and visualization of RNA-seq data from TCGA-STAD and associated clinical information from the TCGA database were analyzed and visualized. Specifically, **Figure 1C** demonstrates that MMD levels were notably elevated in stages T2, T3, and T4 compared to those in stage T1.

### 3.3 MMD is strongly linked to the prognosis of GC

For GEPIA2, we anticipated a correlation between MMD levels and GC prognosis, revealing that patients with high MMD levels had a substantially shorter overall survival time than those with low MMD levels (p=0.028) (**Figure 1F**).

Additionally, KM plot analysis indicated that patients with elevated MMD levels experienced inferior outcomes in terms of overall survival (**Figure 1G**). Furthermore, individuals with high MMD levels had poorer outcomes in terms of disease first progression (**Figure 1H**), suggesting that high MMD expression may be a prognostic risk factor for GC patients.

**Figure 2:**
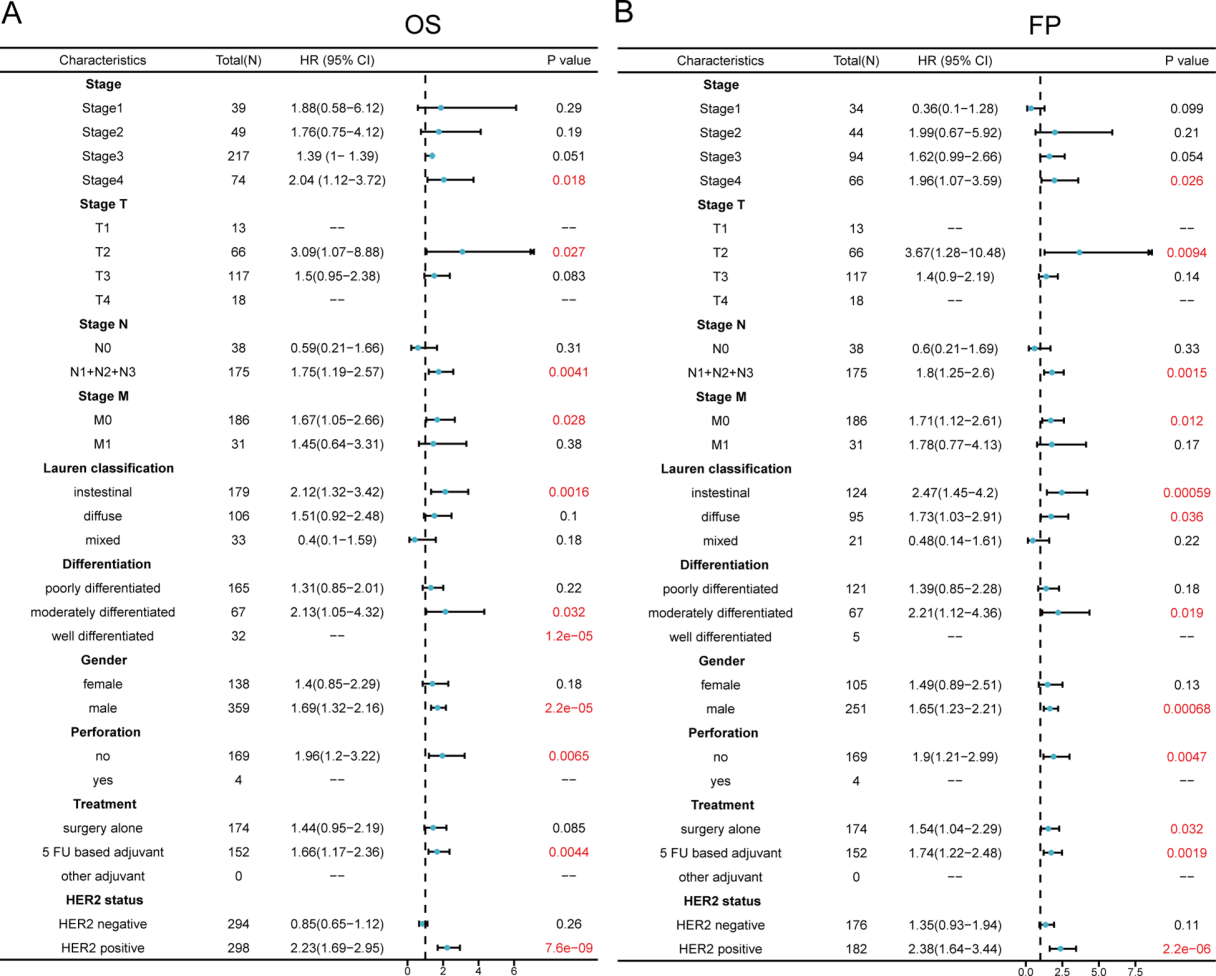
Comparison of prognosis between groups with high and low MMD in clinicopathologic characteristic subgroups in GC. **(A)** Forest plot for OS analysis; **(B)** Forest plot for FP analysis. (OS, Overall survival; FP, First progression).

We examined how MMD expression levels correlated with various clinicopathological characteristics of gastric cancer, including clinical stage, Lauren classification, tumor differentiation, patient sex, gastric perforation, clinical treatment, and human epidermal growth factor receptor 2 (HER2) expression with respect to overall survival (OS) (**Figure 2A**)and first progression (FP) (**Figure 2B**) in gastric cancer patients. The findings indicated a strong association between elevated MMD levels and stage 4 disease in the OS and FP assessments. MMD expression was also significantly correlated with clinical T-stage, N-stage, and M-stage. According to Lauren’s categorization, elevated MMD levels were associated with unfavorable outcomes in intestinal-type GC in both OS and FP assessments (p<0.01). Examination of tumor differentiation revealed a correlation between elevated MMD levels and unfavorable outcomes in patients with moderately differentiated gastric cancer (p<0.05). In the analyses of OS and FP, the prognosis of male patients was more strongly correlated with MMD expression than that of female patients. High MMD levels were associated with worse prognosis in males (p<0.001). Additionally, MMD expression levels affect patient outcomes and disease progression in various clinical treatments. Patients with elevated MMD levels had shorter OS and FP rates when receiving adjuvant chemotherapy with 5-Fluorouracil (5-FU) (p<0.01). Moreover, high MMD levels were linked to deterioration at the time of first disease progression among patients who underwent surgical resection alone. HER2 is a standard tumor marker for GC and is used for prognosis prediction and selection of adjuvant therapeutic agents for GC. As shown in **Figure 2**, HER2-positive individuals with elevated MMD levels experienced decreased overall survival and accelerated disease progression (hazard ratio>2, p<0.00001) compared with those with lower MMD levels. Thus, MMD may be a tumor marker that can assist in determining the prognosis of GC patients.

### 3.4 A significant increase in MMD levels is an independent risk factor for GC

**Figure 3:**
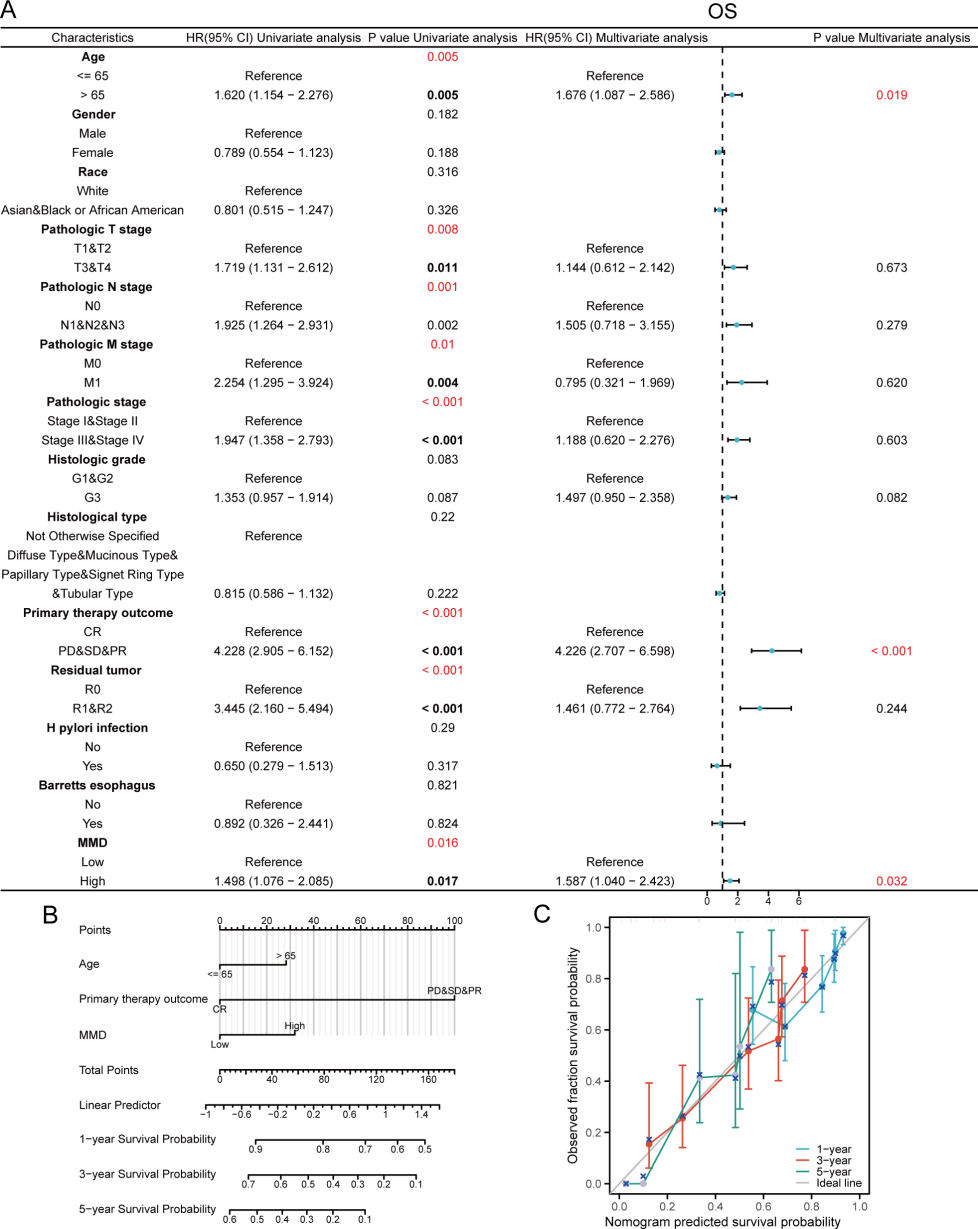
Cox regression analysis on OS. **(A)** Forest plot; **(B)** Prognostic nomogram graph; **(C)** Prognostic calibration curve. (OS, Overall survival).

Cox regression analyses were conducted using MMD expression data from TCGA-STAD and the clinical details of the GC samples. **Figure 3A** demonstrates that in the univariate Cox regression analyses, age, clinical stage, chemotherapy effectiveness, residual tumor size postoperatively, and MMD expression level had a remarkable impact on OS in GC patients. In multifactorial Cox regression analysis, patient age (hazard ratio=1.676, p=0.019), preoperative chemotherapy effect (hazard ratio=4.226, p<0.001), and MMD levels (hazard ratio=1.587, p=0.032) significantly affected the OS of GC patients. Nomograms were developed to predict the chances of survival at 1, 3, and 5 years in GC patients by integrating MMD levels and clinical characteristics (**Figure 3B**). In addition, we evaluated the calibration of each model using calibration plots. Calibration plots indicated that the nomogram was calibrated correctly (**Figure 3C**). Moreover, elevated MMD levels were strongly associated with unfavorable disease-specific survival results in individuals with GC (**Supplementary Figure 1**). Therefore, high MMD expression is an independent risk factor for a poor prognosis in GC patients.

### 3.5 Functional enrichment analysis of MMD

**Figure 4:**
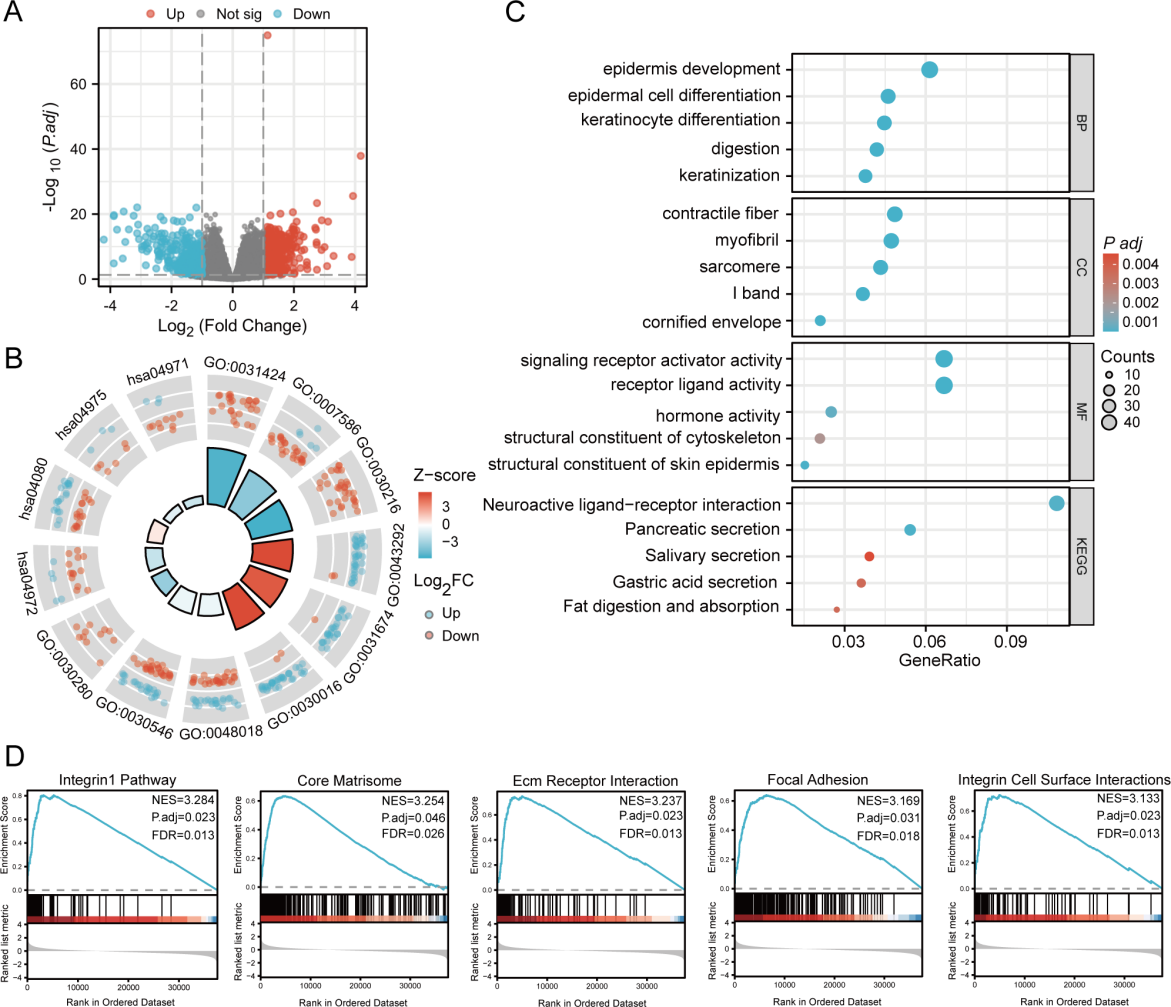
Enrichment analysis. **(A)** Volcano plot for DEGs; **(B)** Circle diagram; **(C)** Bubble plot; **(D)** GSEA analysis. (GO, Gene Ontology; BP, Biological process; CC, Cellular component; MF, Molecular function; KEGG, Kyoto Encyclopedia of Genes and Genomes; GSEA, Gene set enrichment analysis).

We compared samples exhibiting high MMD expression (n=188) and samples with low MMD expression (n=187) obtained from TCGA (**Supplementary Table 2**). Results indicated the presence of 815 DEGs, comprising 443 genes with increased expression and 372 genes with decreased expression (**Figure 4A**). We performed the GO and KEGG analyses to gain an overview of such functions. Numerous pathways were enriched, including digestion, differentiation of epidermal cells, activation of signaling receptors, secretion of pancreatic enzymes, digestion and absorption of fats, interactions between neuroactive ligands and receptors, and secretion of gastric acid (**Figures 4B, C**).

In addition, we conducted a GSEA of MMD. **Figure 4D** depicts the five highest-ranked pathways sorted by normalized enrichment score (NES). The results indicated significant enrichment of the integrin1 pathway, core matrisome, integrin cell-surface interactions, focal adhesion, and extracellular matrix (ECM) receptor interaction.

### 3.6 MMD is associated with the tumor immune microenvironment in GC

**Figure 5:**
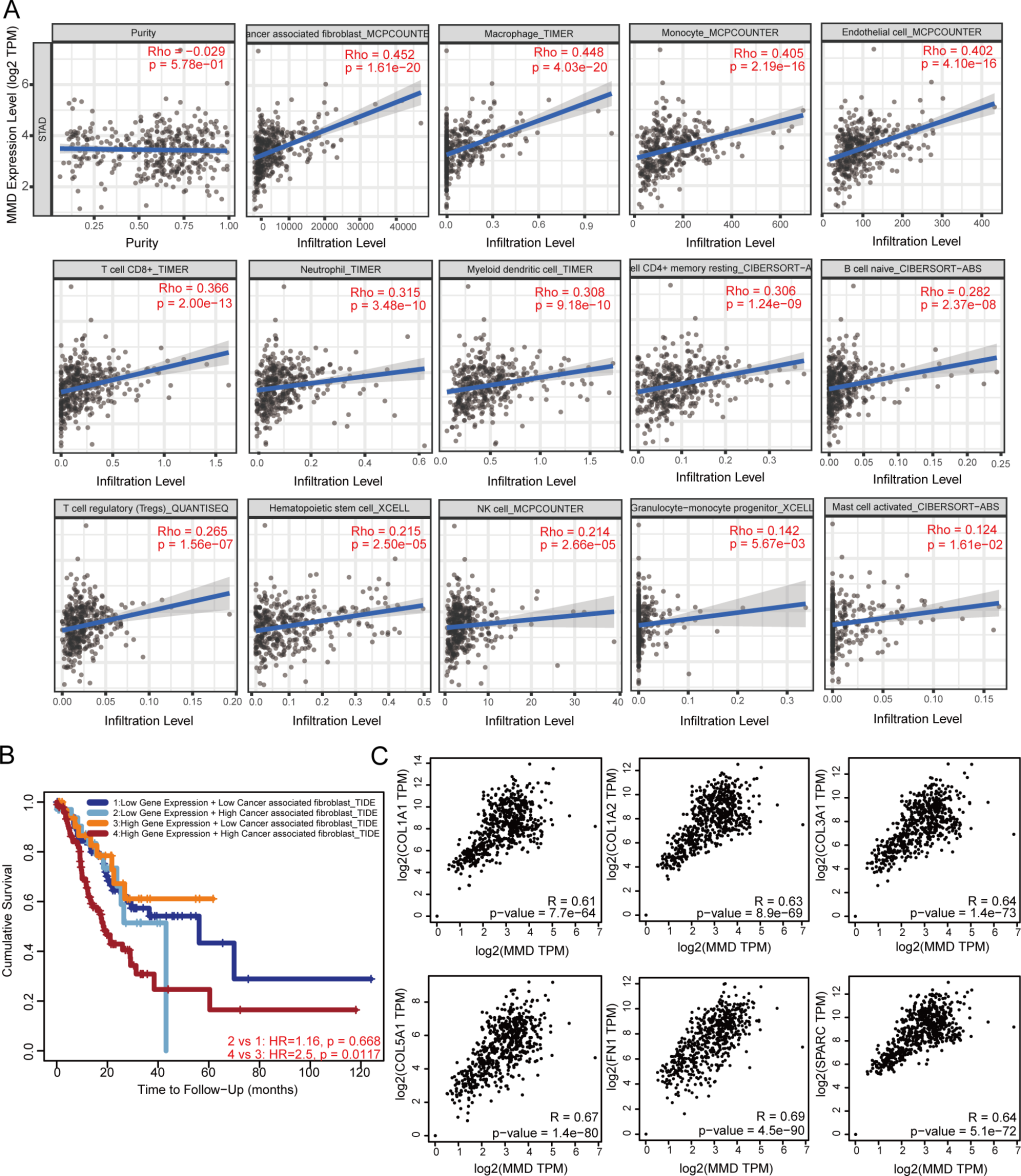
MMD and the immune microenvironment of GC. **(A)** Correlation analysis in GC; **(B)** Impact of MMD and CAFs on the prognosis of GC patients; **(C)** Correlation analysis of MMD with six markers of CAFs in GC. (CAFs, Cancer-associated fibroblasts).

As shown in **Figure 5A**, the data revealed a correlation between MMD levels and the presence of different immune cells, such as cancer-associated fibroblasts, monocytes, and macrophages, in GC (p<0.05).

Further analyses were performed on MMD and cancer-associated fibroblasts. As illustrated in **Figure 5B**, patients displaying high levels of MMD expression were found to have a poorer prognosis when associated with high levels of cancer-associated fibroblast infiltration than those exhibiting lower levels of such infiltration (p=0.0117). Correlation analysis of MMD with six important markers of gastric cancer-related fibroblasts (CAFs), including collagen type I alpha 1 chain (COL1A1), collagen type I alpha 2 chain (COL1A2), collagen type III alpha 1 chain (COL3A1), collagen type V alpha 1 chain (COL5A1), fibronectin 1 (FN1), and secreted protein acidic and cysteine rich (SPARC), was carried out. The correlations between MMD and these genes were above 0.6 with a p-value < 0.00001 (**Figure 5C**).

### 3.7 MMD promotes the growth of GC cells

**Figure 6:**
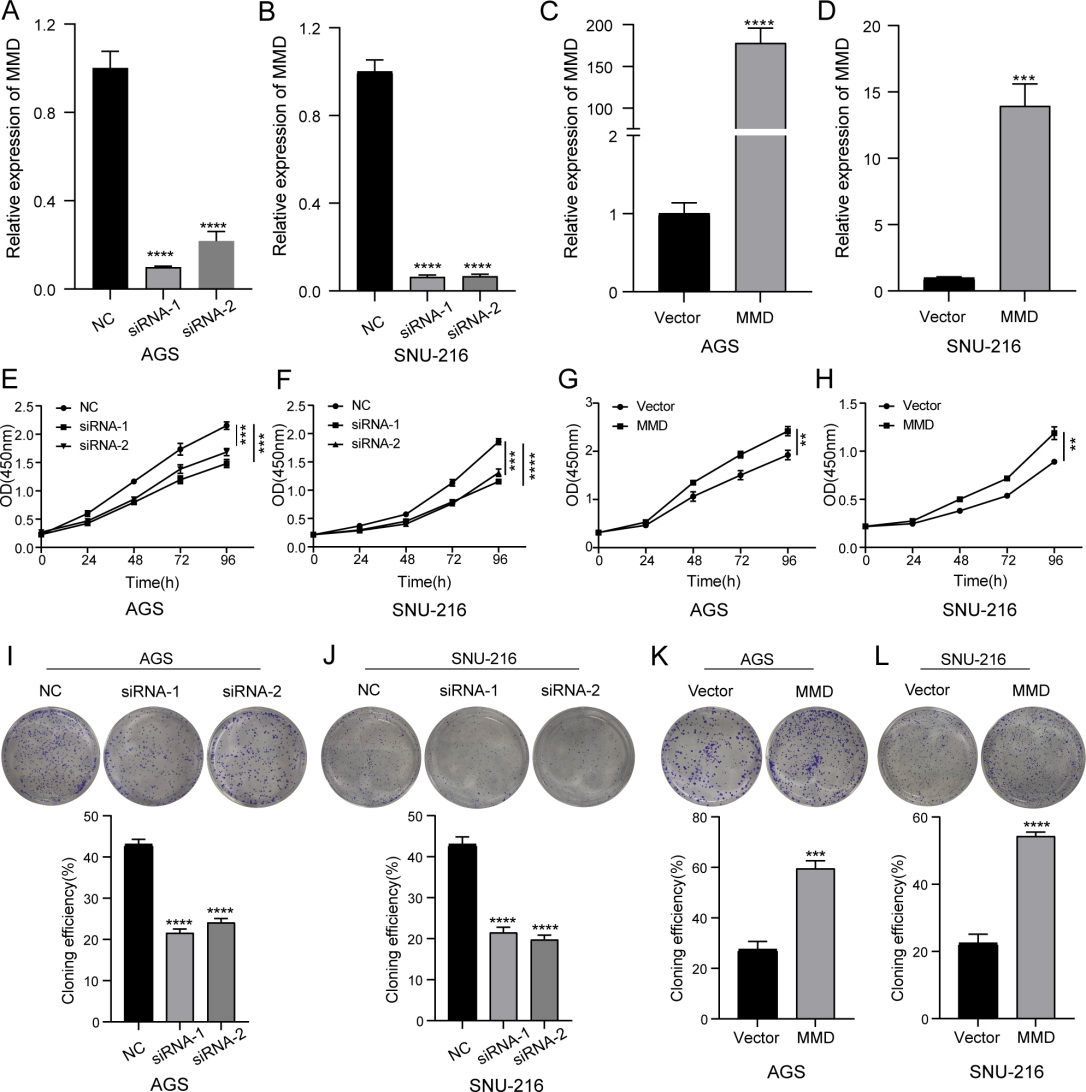
CCK-8 and clone formation assay. **(A–D)** Validation of the effectiveness of siRNA and overexpression plasmid in AGS and SNU-216 cells; **(E, F)** Down-regulation of MMD expression slows down the proliferation rate; **(G, H)** MMD overexpression accelerates the proliferation rate; **(I, J)** Down-regulation of MMD expression attenuates the proliferation ability; **(K, L)** MMD overexpression enhances the proliferation ability. (**p<0.01, ***p<0.001, ****p<0.0001).

To assess the effect of altered MMD expression on the growth rate of GC cells, we measured cell proliferation using the CCK-8 assay. Cell proliferation charts were created using optical density (450 nm) at five different time intervals (0h, 24h, 48h, 72h, and 96h). The findings indicated that reducing MMD expression in the group resulted in a notable decrease in the cell proliferation rate compared to the NC group in AGS and SNU-216, suggesting that inhibiting MMD expression could slow down the growth of gastric cancer cells (**Figures 6E, F**). **Figures 6G, H** demonstrated that MMD overexpression led to a higher proliferation rate in AGS and SNU-216 cells than in the control group (Vector).

Next, we examined the effect of MMD on the proliferative capacity of gastric cancer cells using a plate clone formation assay. **Figures 6I, J** demonstrate a decrease in the clone formation rate of GC cells following the downregulation of MMD level, suggesting that reducing MMD levels in GC cells weakened their proliferation capacity compared with that in the NC group. Moreover, MMD overexpression enhanced the clone formation ability of gastric cancer cells (**Figures 6K, L**).

**Figure 7:**
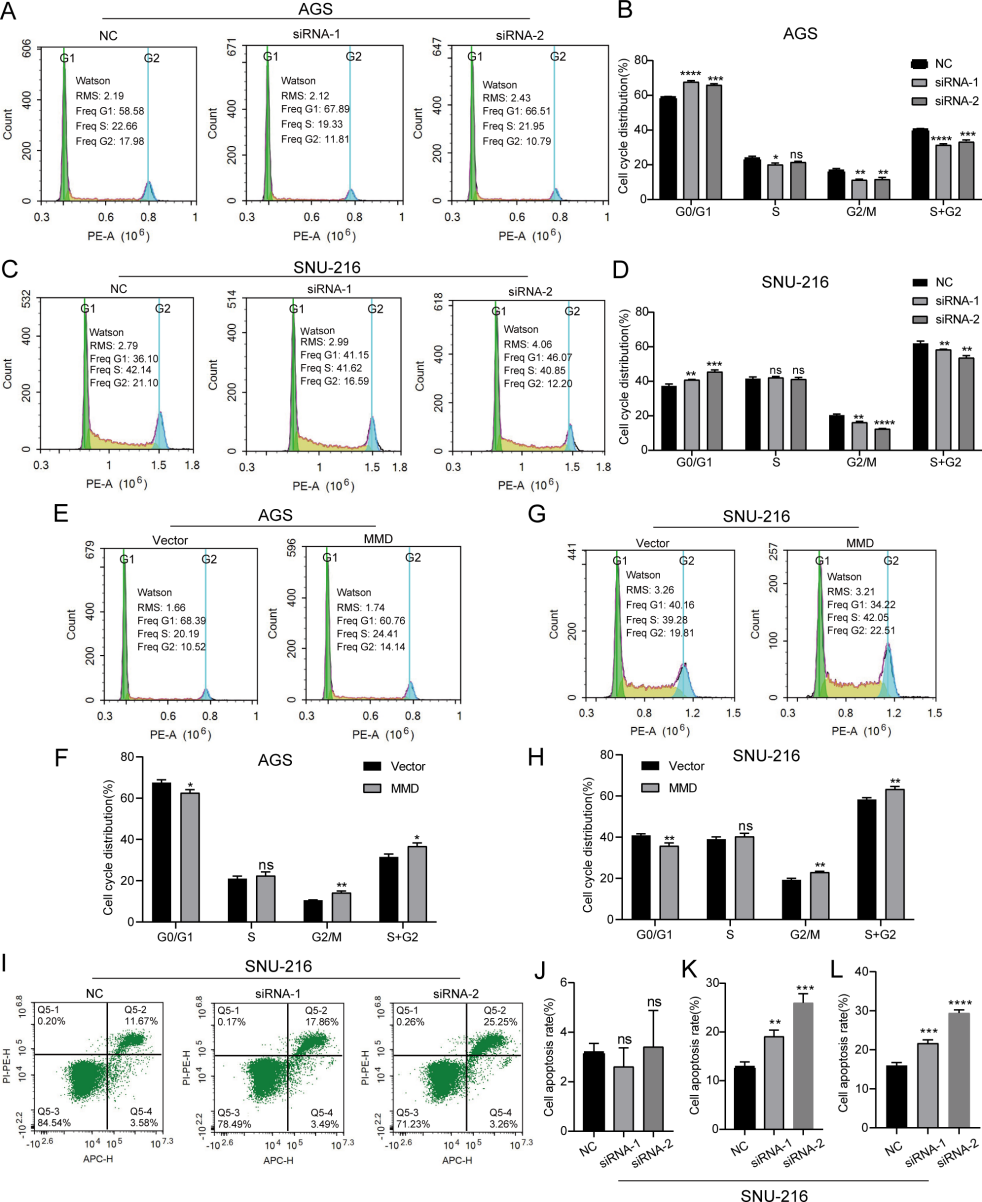
Cell cycle and apoptosis assays. **(A, B)** Down-regulation of MMD expression inhibits AGS cell growth; **(C, D)** Down-regulation of MMD expression inhibits SNU-216 cell growth; **(E, F)** MMD overexpression promotes AGS cell growth; **(G, H)** MMD overexpression promotes SNU-216 cell growth; **(I)** Down-regulation of MMD expression promotes SNU-216 cell apoptosis; (J–L) Early, middle-late, and total apoptosis in SNU-216 cells. ns: not significant (p>0.05). *p<0.05, **p<0.01, ***p<0.001, ****p<0.0001.

Flow cytometry was used to examine the cell cycle and apoptosis of gastric cancer cells with altered MMD expression. In AGS cells, the study findings indicated that the cells in the G0/G1 phase were significantly higher, and the cells in the G2/M and S+G2 phases were all decreased in the MMD expression down-regulation group (**Figures 7A, B**). In SNU-216 cells, the cell cycle assay results of the MMD low expression group were similar to those of AGS cells (**Figures 7C, D**). This suggests that the downregulation of MMD levels may slow the growth of GC cells by blocking cells in the G0/G1 phase. Furthermore, following the increase in MMD expression, a notable reduction in G0/G1 phase cells, an insignificant alteration in S phase cells, and an increase in G2/M and S+G2 phase cells were observed in the MMD overexpression group (**Figures 7E–H**). The apoptosis assay of SNU-216 cells revealed a higher percentage of total apoptotic cells in the group with reduced MMD expression than in the NC group. This was mainly due to the higher rate of late apoptotic cells (**Figures 7I–L**).

### 3.8 Down-regulation of MMD inhibits GC cell growth *in vivo*

**Figure 8:**
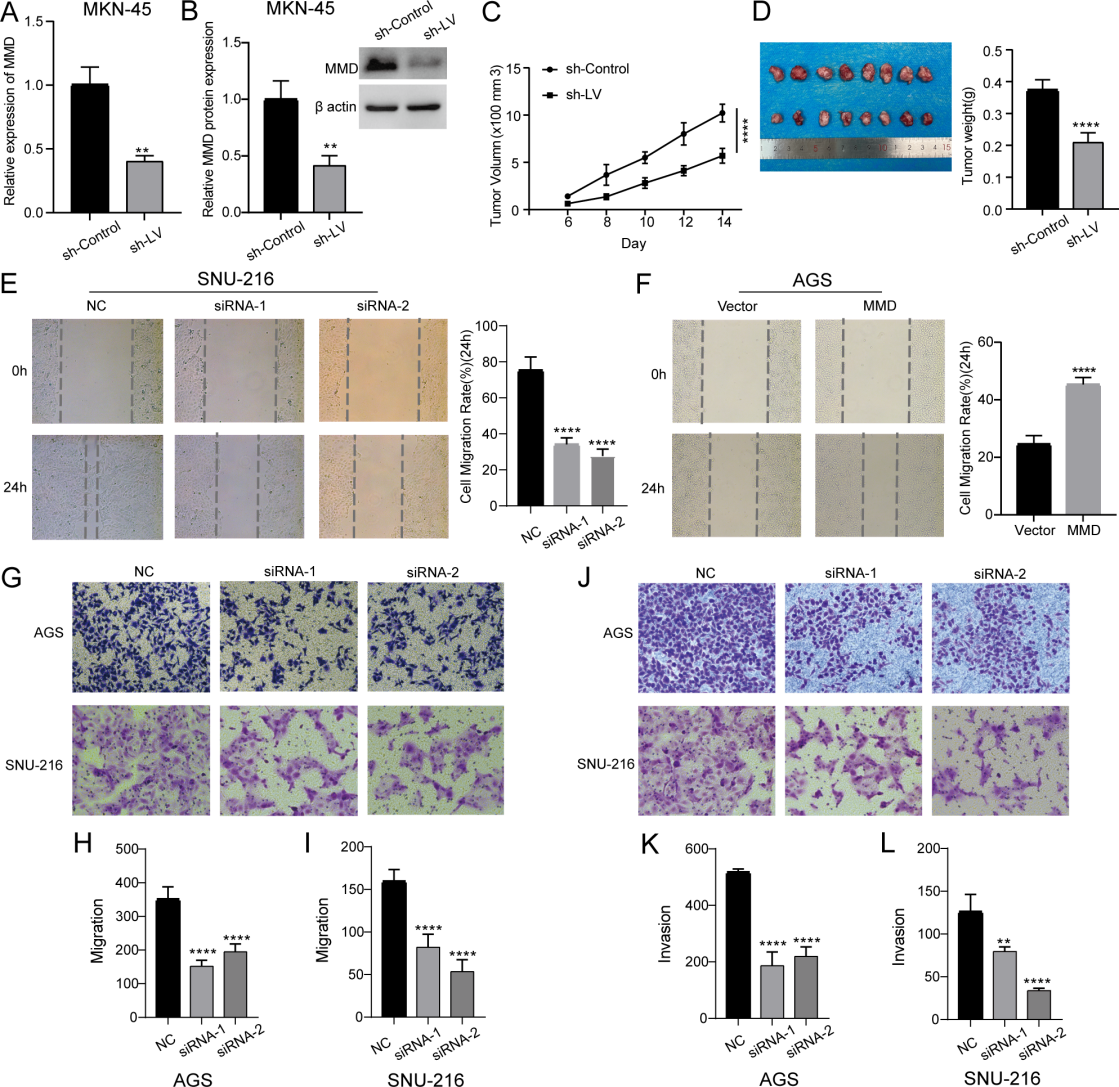
Animal experiments, scratch assay, migration, and invasion experiments. **(A, B)** Construction of lentiviral stable cell line by MMD knockdown in MKN-45 cells (qPCR and western blotting); **(C, D)** Subcutaneous tumor formation experiment in nude mice; **(E)** Down-regulation of MMD expression level inhibits wound healing in SNU-216 cells; **(F)** MMD overexpression promotes wound healing in AGS cells; **(G–I)** Down-regulation of MMD expression inhibits migration; **(J–L)** Down-regulation of MMD expression inhibits invasion. (**p<0.01, ****p<0.0001).

We constructed a stable cell line by lentiviral-mediated MMD knockdown in MKN-45 cells (**Figures 8A, B**). As shown in **Figure 8C**, the growth rate of subcutaneous graft tumors in the MMD knockdown group (sh-LV) was slower than that in the control group (sh-Control). Simultaneously, it was evident that the tumor size in the MMD knockdown group (sh-LV) was notably smaller than that in the sh-Control group (**Figure 8D**). Hence, the results indicated that MMD knockdown slowed tumor growth *in vivo* caused by MKN-45.

### 3.9 MMD stimulates the migration and invasion of GC cells

**Figure 9:**
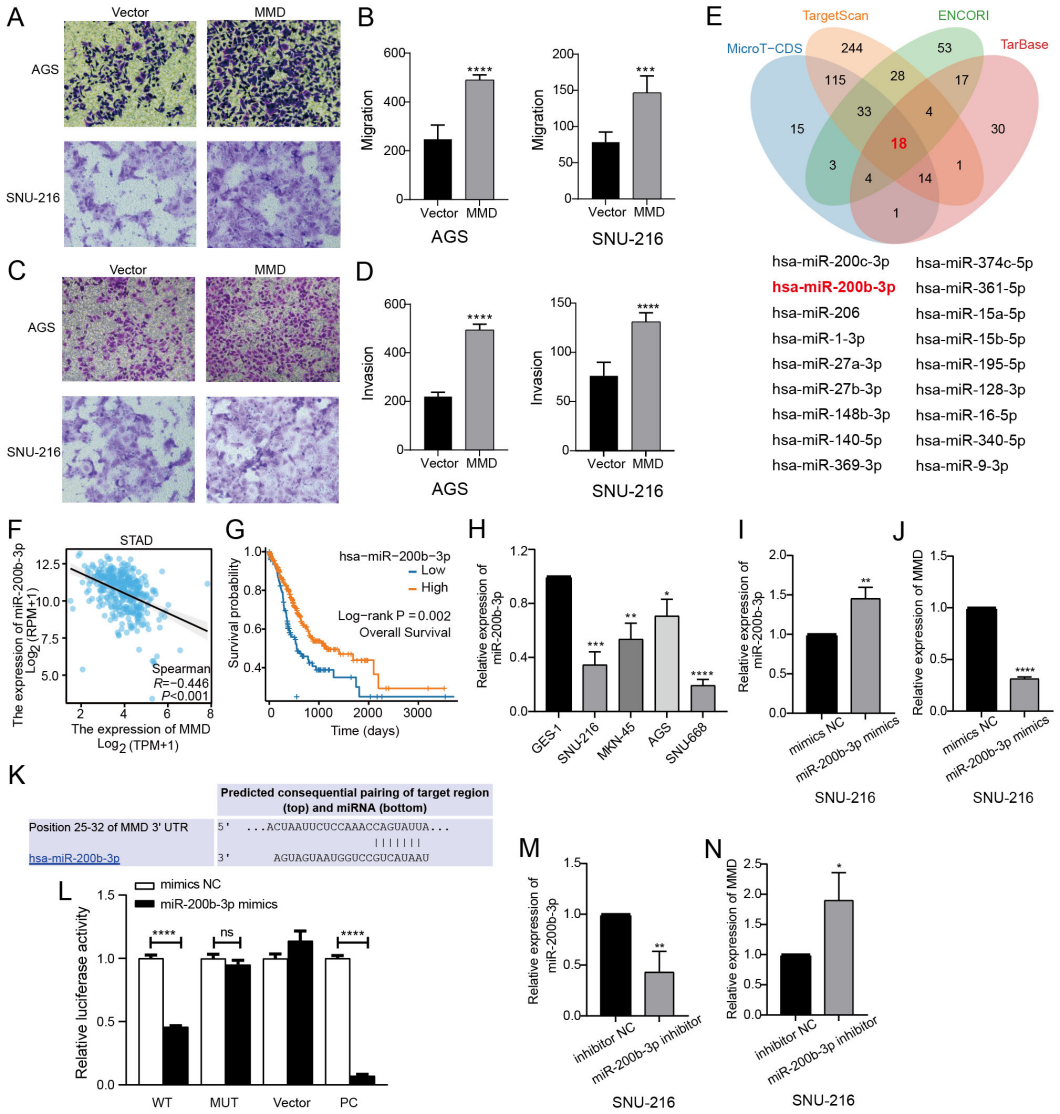
MMD overexpression affects GC cell invasion and migration and miR-200b-3p regulates MMD expression. **(A–D)** Transwell assay of MMD overexpression in AGS and SNU-216; **(E)** Predicted miRNAs from four databases were taken to the intersection (Venn diagram); **(F)** Correlation analysis of miR-200b-3p and MMD in GC; **(G)** Impact of miR-200b-3p expression on OS in GC; **(H)** miR-200b-3p expression in GC cells (qPCR); **(I, M)** Validation of the validity of miR-200b-3p mimics and inhibitor; **(K)** Predicted binding sites of miR-200b-3p and MMD; **(L)** Dual luciferase assay; **(J, N)** miR-200b-3p regulation of MMD expression. (ns: p>0.05, *p<0.05, **p<0.01, ***p<0.001, ****p<0.0001) (OS, Overall survival).

**Figure 8E** shows that the group with decreased MMD expression had a slower scratch healing rate and reduced 24-hour cell migration rate compared with the NC group. Following the reduction in MMD levels in the AGS and SNU-216 cell migration tests, there was a notable decrease in the number of cells moving across the chamber membrane to access the exterior lower surface of the chamber compared with that in the control group at the corresponding time (**Figures 8G–I**). During the invasion trial, there was a notable decrease in the number of cells that migrated through the stromal gel in the MMD-knockdown group compared with that in the NC group (**Figures 8J–L**). The results of the three tests showed that suppression of MMD expression hindered GC cell movement and infiltration activities. In addition, as shown in **Figure 8F**, the scratch-healing speed was significantly faster in the MMD overexpression group. The results of the Transwell assay showed that MMD overexpression accelerated GC cell migration (**Figures 9A, B**) and invasion (**Figures 9C, D**).

### 3.10 miR-200b-3p negatively regulates MMD level in GC cells

The miRNAs upstream of MMD were predicted using four databases (MicroT-CDS, TargetScan, ENCORI, and TarBase). Eighteen miRNAs were identified by taking the intersection of the four prediction results (**Figure 9E**), which correlated with MMD expression in GC tissues (**Supplementary Figure 2**). Among these miRNAs, miR-200b-3p exhibited the strongest negative correlation with MMD expression (p<0.001) (**Figure 9F**). **Figure 9G** showed a strong correlation between low miR-200b-3p levels and poor overall survival in individuals with GC. qPCR confirmed that miR-200b-3p expression was low in the GC cells (**Figure 9H**). Our findings indicated that MMD expression decreased with increasing miR-200b-3p levels (**Figures 9I, J**), and conversely, MMD expression increased with decreasing miR-200b-3p levels (**Figures 9M, N**). Dual-luciferase assay confirmed that MMD was a direct target of miR-200b-3p (**Figures 9K, L**).

## 4 Discussions

Early detection and proper treatment of stomach cancer can extend the lifespan of individuals with GC. The identification of efficient biomarkers is essential for the diagnosis and prognosis of GC. Our study found a notable increase in MMD expression in GC using TCGA-STAD and GTEx datasets. Elevated MMD expression was associated with poor survival. Further examination using the KM plot database indicated a correlation between elevated MMD levels and unfavorable outcomes in individuals with GC, including age, sex, pathological stage, Lauren classification, treatment, and HER2 status. Additionally, Cox regression analysis suggested that high MMD expression was an independent risk factor for poor GC prognosis. Functional enrichment analysis suggested that the genes associated with MMD expression were mainly enriched in the integrin 1 pathway, core matrisome, and ECM receptor interaction pathways. Studies showed that integrin β1 regulated diverse functions including proliferation, apoptosis, migration, invasion, angiogenesis, and drug resistance in cancerous cells (17–21). The formation of fibrils in the ECM within the tumor microenvironment is crucial for tumor metastases (22). MMD has also been linked to CAFs in GC. CAFs are present within the tumor microenvironment and are involved in tumor progression and spread, drug resistance, and evasion of the immune system (23–25). Therefore, MMD may be involved in cancer development.

Experimental validation demonstrated a remarkable increase in MMD levels in GC cells. The proliferation rate and ability of the downregulated MMD group was notable decreased compared with those of the NC group. Flow cytometry revealed cell arrest in the MMD knockdown group at the G0/G1 phase and conspicuous apoptosis. Following MMD repression, wound healing and transwell assays revealed significant curtailment of GC cell migration and invasion potency. In contrast, an increase in MMD levels in GC cells boosted cell growth, notably improving cell migration and invasion capabilities. These findings indicated that MMD acts as a pro-carcinogenic gene in GC cells, promoting their proliferation, migration, and invasion.

Reports have linked miR-200b-3p to the progression of various tumors, including breast, colorectal, esophageal, gastric, liver, lung, and prostate cancer (26–32). However, no studies have linked miR-200b-3p expression to MMD. In this study, miR-200b-3p was selected based on correlation and prognostic analyses. Subsequently, it was experimentally verified that miR-200b-3p had low expression in GC cells and negatively regulated MMD expression. The dual-luciferase assay results showed that miR-200b-3p directly targeted MMD.

Our research utilized bioinformatics tools to predict MMD level and its prognostic importance in GC and anticipate its possible roles. Our study confirmed the high expression of MMD in GC cells, revealing its influence on cell biology. We also identified miR-200b-3p as a direct upstream regulatory molecule in MMD. Therefore, MMD should be examined in greater depth in future studies.

## 5 Conclusions

In summary, GC cells exhibit high MMD expression. High MMD expression increased poor prognosis of GC patients and was a standalone risk factor for unfavorable outcomes. Various studies have indicated that high MMD expression enhances growth, migration, and invasion of GC cells. Furthermore, MMD was suppressed by miR-200b-3p, a direct target gene. MMD may serve as a novel indicator for diagnosing and predicting stomach cancer outcomes.

## Data Availability

The original contributions presented in the study are included in the article/**Supplementary Material**. Further inquiries can be directed to the corresponding authors.
