# Correction: Identification and validation of monocyte to macrophage differentiation-associated as a prognostic biomarker in gastric cancer

**DOI:** 10.3389/fonc.2025.1649116

**Published:** 2025-06-27

**Authors:** Suyang Bai, Zhaofeng Chen, Rui Ji, Yuping Wang, Yongning Zhou, Qinghong Guo, Liang Qiao

**Affiliations:** ^1^ The First Clinical Medical College, Lanzhou University, Lanzhou, China; ^2^ Department of Gastroenterology, The First Hospital of Lanzhou University, Lanzhou, China; ^3^ Gansu Province Clinical Research Center for Digestive Diseases, The First Hospital of Lanzhou University, Lanzhou, China; ^4^ Storr Liver Centre, The Westmead Institute for Medical Research (WIMR), The University of Sydney, Westmead, NSW, Australia

**Keywords:** MMD, stomach neoplasms, prognosis, biomarker, miR-200b-3p

Affiliations 1, 2 and 3 were omitted for author Liang Qiao. These affiliations have now been added for author Liang Qiao.

The original version of this article has been updated.

