# Supplementary material for: Identification and validation of monocyte to macrophage differentiation-associated as a prognostic biomarker in gastric cancer
Source: Front Oncol. 2025 Apr 16;15:1508355. doi: 10.3389/fonc.2025.1508355 (PMC12040639; doi:10.3389/fonc.2025.1508355)
Supplement: Supplementary file 1 [file Table1.docx]

**Supplementary Tables**

**Table S1.** qPCR primer sequence

| Name |  | Primer sequence (5' → 3') |
| --- | --- | --- |
| MMD | Forward | CACCGATGGACTTCAGGAACTTG |
|  | Reverse | CAGATGGCGTGGGCAAATGG |
| β-actin | Forward | TGGCACCCAGCACAATGAA |
|  | Reverse | CTAAGTCATAGTCCGCCTAGAAGCA |
| miR-200b-3p | Forward | GCTGCTGAATTCCATCTAATTTCCAAAAG |
|  | Reverse | Universal primer (TaKaRa, 638313) |
| U6 | Forward | TaKaRa, 638313 |
|  | Reverse | TaKaRa, 638313 |

**Table S2.** MMD and clinical features of gastric cancer.

| Characteristics | Low expression of MMD | High expression of MMD | *p* value |
| --- | --- | --- | --- |
| n | 187 | 188 |  |
| Age, n (%) |  |  | 0.982 |
| <= 65 | 81 (21.8%) | 83 (22.4%) |  |
| > 65 | 102 (27.5%) | 105 (28.3%) |  |
| Gender, n (%) |  |  | 0.410 |
| Male | 124 (33.1%) | 117 (31.2%) |  |
| Female | 63 (16.8%) | 71 (18.9%) |  |
| Pathologic T stage, n (%) |  |  | **0.022** |
| T1 | 14 (3.8%) | 5 (1.4%) |  |
| T2 | 45 (12.3%) | 35 (9.5%) |  |
| T3 | 87 (23.7%) | 81 (22.1%) |  |
| T4 | 40 (10.9%) | 60 (16.3%) |  |
| Pathologic N stage, n (%) |  |  | 0.793 |
| N0 | 59 (16.5%) | 52 (14.6%) |  |
| N1 | 48 (13.4%) | 49 (13.7%) |  |
| N2 | 39 (10.9%) | 36 (10.1%) |  |
| N3 | 34 (9.5%) | 40 (11.2%) |  |
| Pathologic M stage, n (%) |  |  | 0.142 |
| M0 | 169 (47.6%) | 161 (45.4%) |  |
| M1 | 9 (2.5%) | 16 (4.5%) |  |
| Pathologic stage, n (%) |  |  | 0.386 |
| Stage I | 30 (8.5%) | 23 (6.5%) |  |
| Stage II | 60 (17%) | 51 (14.5%) |  |
| Stage III | 69 (19.6%) | 81 (23%) |  |
| Stage IV | 17 (4.8%) | 21 (6%) |  |
| Histologic grade, n (%) |  |  | 0.488 |
| G1 | 5 (1.4%) | 5 (1.4%) |  |
| G2 | 74 (20.2%) | 63 (17.2%) |  |
| G3 | 104 (28.4%) | 115 (31.4%) |  |

**Supplementary Figures**

**
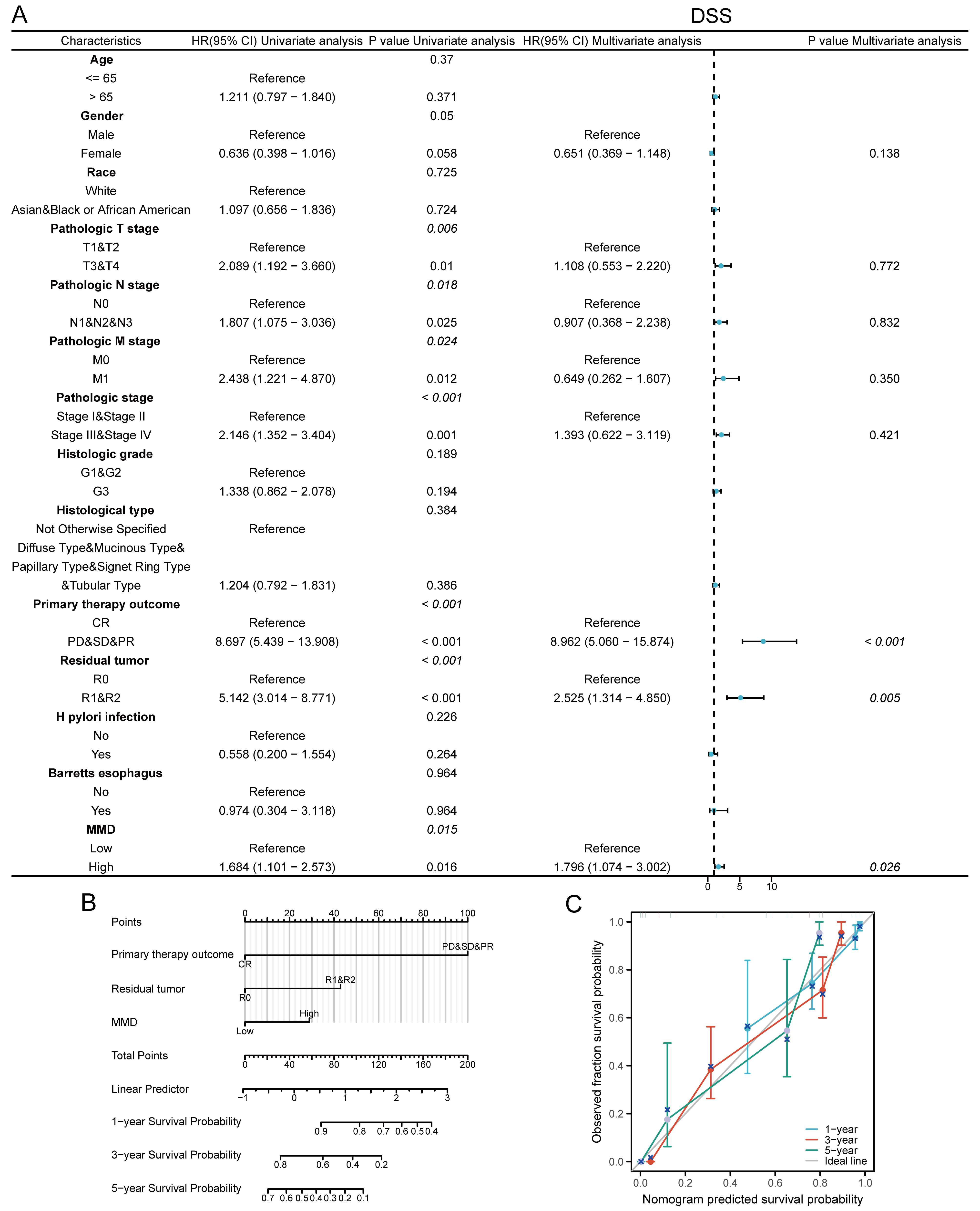
**

**Fig. S1.** Cox regression analysis on DSS. (A) Forest plot; (B) Prognostic nomogram graph; (C) Prognostic calibration curve. (DSS: Disease specific survival)


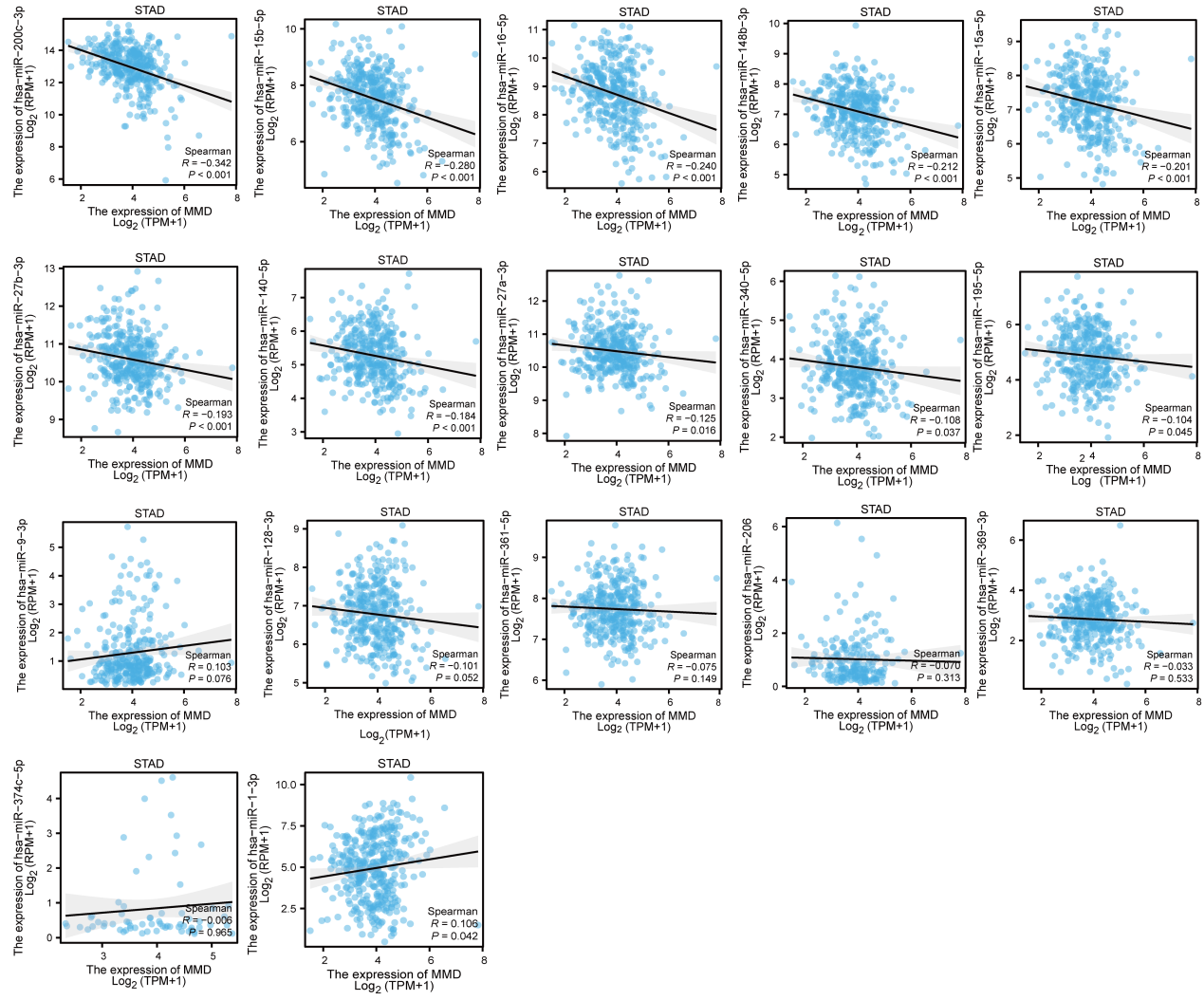


**Fig. S2.** Correlation analysis of 17 miRNAs with MMD in GC.
