# Supplementary material for: Identification and validation of monocyte to macrophage differentiation-associated as a prognostic biomarker in gastric cancer
Source: Front Oncol. 2025 Apr 16;15:1508355. doi: 10.3389/fonc.2025.1508355 (PMC12040639; doi:10.3389/fonc.2025.1508355)
Supplement: Supplementary file 2 [file Table2.docx]

**Abbreviations**

| TCGA | The Cancer Genome Atlas |
| --- | --- |
| STAD | Stomach adenocarcinoma |
| GTEx | Genotype-Tissue Expression |
| GC | Gastric cancer |
| MMD | Monocyte to macrophage differentiation-associated |
| EMT | Epithelial-mesenchymal transition |
| Ras | Rat sarcoma protein |
| ERK | Extracellular regulated protein kinases |
| AKT | Protein kinase B |
| ACSL4 | Acyl-CoA synthetase long chain family member 4 |
| MBOAT7 | Membrane bound O-acyltransferase domain containing 7 |
| DEGs | Differentially expressed genes |
| GO | Gene Ontology |
| KEGG | Kyoto Encyclopedia of Genes and Genomes |
| GSEA | Gene set enrichment analysis |
| qPCR | Real-time quantitative PCR |
| shRNA | Short hairpin RNA |
| OS | Overall survival |
| FP | First progression |
| HER2 | Human epidermal growth factor receptor 2 |
| 5-FU | 5-Fluorouracil |
| NES | Normalized enrichment score |
| ECM | Extracellular matrix |
| CAFs | Cancer-related fibroblasts |
| COL1A1 | Collagen type I alpha 1 chain |
| COL1A2 | Collagen type I alpha 2 chain |
| COL3A1 | Collagen type III alpha 1 chain |
| COL5A1 | Collagen type V alpha 1 chain |
| FN1 | Fibronectin 1 |
| SPARC | Secreted protein acidic and cysteine rich |
